# Samuel Cartwright

**Published:** 1891-09

**Authors:** 


### Obituary.

SAMUEL CARTWRIGHT, F. R. C. S.—With extreme regret we have to record the death of Mr. Samuel Cartwright, who passed to his rest on August 23rd, at the age of 76. The son of a man celebrated in the dental profession, Mr. Cartwright, himself, followed in his footsteps and established for himself a reputation in the profession of his choice, while he earned the respect and affection of all those who had the privilege of being associated with him. He studied at the London Hospital, taking his M. R. C. S. in 1838 and his F. R. C. S., in 1859. He commenced his professional work as a dentist in Sackville Street, whence he removed to Old Burlington Street to undertake the practice which his father's failing health obliged him to give up. In those days the profession was not what it is now, and many and severe were the struggles through which it had to go before its final establishment by the Dentist's Acts, and formation of the Odontological Society, and, subsequently, of the British Dental Association. Mr. Cartwright's probity of character and high professional attainments made him a frequent referee upon professional matters. When the London Dental Hospital was initiated Mr. Cartwright was one of its first surgeons, while he undertook for the school associated with it to lecture upon Dental Surgery and Pathology; he was also, for many years, treasurer to the Hospital. Mr. Cartwright was appointed Dental Surgeon also to the King's College Hospital, and, subsequently, Examiner at the Dental Board of the Royal College of Surgeons, giving much satisfaction in each post. Later in life, owing to great pressure of work, he retired from these positions. Mr. Cartwright was distinguished by the honor and uprightness of his character and the value of his friendship was greatly appreciated by those who shared it. We must add that Mr. Cartwright was the first to occupy the specially-instituted Chair of Professor of Dental Surgery at King' College, and upon two occasions received the Blue Riband of the Dental Profession, election to the Presidential Chair of the Odontological Society, again following in the steps of his father who was the first to fill the honored post. The social side of Mr. Cartwright's character was conspicuous by his love for music, having been one of the original members of the "Round Catch and Cannon Club," as well as his no mean attainments with the pencil and brush. After a blameless and distinguished life Mr. Cartwright has left us with the legacy of a well-beloved memory. Many who are now busy practitioners will remember how, in the old "Soho days," Mr. Cartwright's kindly bearing endeared him to the students, for whom he undertook the presidency of their Society, as well as being the chairman at the first dinner given by the Present and Past Students of the London Dental Hospital.—*London Dental Record.*

